# Long-term stimulation by implanted pacemaker enables non-atrophic treatment of bilateral vocal fold paresis in a human-like animal model

**DOI:** 10.1038/s41598-024-60875-0

**Published:** 2024-05-07

**Authors:** Kassandra Walluks, Bianca Hoffmann, Carl-Magnus Svensson, Gerhard Förster, Andreas H. Müller, Jonathan Jarvis, Justin Perkins, Marc Thilo Figge, Dirk Arnold

**Affiliations:** 1 Applied Systems Biology, Leibniz Institute for Natural Product Research and Infection Biology-Hans Knöll Institute, Jena, Germany; 2 Faculty of Biological Sciences, Friedrich Schiller University Jena, Jena, Germany; 3 Institute of Zoology and Evolutionary Research, Faculty of Biological Sciences, Friedrich Schiller University Jena, Jena, Germany; 4 Clinic for Otorhinolaryngology/Plastic Surgery, Wald-Klinikum Gera, Gera, Germany; 5 Faculty of Science, Sport and Exercise Sciences, John Moores University, Liverpool, UK; 6 Royal Veterinary College London, London, UK; 7 Institute of Microbiology, Faculty of Biological Sciences, Friedrich Schiller University Jena, Jena, Germany; 8 Clinic and Polyclinic for Otorhinolaryngology, University Hospital Jena, Jena, Germany

**Keywords:** Immunohistochemistry, Image processing, Respiratory signs and symptoms

## Abstract

A wide variety of treatments have been developed to improve respiratory function and quality of life in patients with bilateral vocal fold paresis (BVFP). One experimental method is the electrical activation of the posterior cricoarytenoid (PCA) muscle with a laryngeal pacemaker (LP) to open the vocal folds. We used an ovine (sheep) model of unilateral VFP to study the long-term effects of functional electrical stimulation on the PCA muscles. The left recurrent laryngeal nerve was cryo-damaged in all animals and an LP was implanted except for the controls. After a reinnervation phase of six months, animals were pooled into groups that received either no treatment, implantation of an LP only, or implantation of an LP and six months of stimulation with different duty cycles. Automated image analysis of fluorescently stained PCA cross-sections was performed to assess relevant muscle characteristics. We observed a fast-to-slow fibre type shift in response to nerve damage and stimulation, but no complete conversion to a slow-twitch-muscle. Fibre size, proportion of hybrid fibres, and intramuscular collagen content were not substantially altered by the stimulation. These results demonstrate that 30 Hz burst stimulation with duty cycles of 40% and 70% did not induce PCA atrophy or fibrosis. Thus, long-term stimulation with an LP is a promising approach for treating BVFP in humans without compromising muscle conditions.

## Introduction

The laryngeal pacemaker (LP) is a device designed to allow patients with bilateral vocal fold paresis (BVFP) an improved inspiration without having their voice affected by the treatment^1–6^.

BVFP is a disease that can result from bilateral injury or transection of the recurrent laryngeal nerve (RLN). This leads to paralysis of the *posterior cricoarytenoid* (PCA) muscle by denervation and, subsequently to the reinnervation phase, to its paresis^7,8^. Synkinetic reinnervations are observed in 21–88% of the cases, depending on the threshold. The left and right PCA are muscles that are located collateral, dorsally at the cricoid cartilage. Their contraction during inhalation results in a movement of the arytenoid cartilages, which abduct the vocal folds to allow the necessary airflow. In paralysis, the vocal folds remain in a paramedian position, passively constricting the glottis when the patient attempts to inhale^4,8^. Patients then suffer from exertional dyspnoea or even resting dyspnoea^4,9^.

A wide variety of therapies has been developed over the decades to treat BVFP. Frequently, these patients require tracheostomy. Additionally, the inspiration can be successfully improved by increasing the glottal gap, e.g. through laterofixation^4,10,11^, surgically selective reinnervation^4,12–14^, partial resections of the arytenoid cartilage, or of the vocal folds themselves by means of laser surgery^4,8,15–17^. Most of these procedures are permanent and impair the voice, as they cause irreversible changes in the vocal folds. Therefore, to improve the patients' lives, a compromise between facilitating breathing and harming the voice is attempted^4,8,18^. The idea of reanimating paralysed or paretic PCAs first emerged in the 1970s. Zealear and Dedo 1977 stimulated the paralysed PCA in dogs electrically^19^. Over the decades, the idea was developed further and experiments were carried out on sheep^20^ and dogs^21–23^. In 1996, the paretic vocal folds of patients were reactivated for the first time^24^. These preliminary works allowed the development of an LP, which we used in the present study to carry out long-term experiments on sheep. This therapeutic approach involves the use of functional electrical stimulation (FES) to stimulate the reinnervated PCA using an electrode inserted into the muscle near the terminal nerve branches. This acts like a temporary laterofixation, effectively dilating the glottis, and enabling a comfortable inspiration without affecting the vocal folds or voice^2–6,9,20,25,26^. Patients would benefit most from LP if the glottis was kept open as long as possible and therefore continuous stimulation with a duty cycle (DC) of 100%, i.e. stimulation over the entire respiratory cycle, could most effectively avoid the feeling of respiratory distress. However, studies have shown that muscles respond differently to internal and external influences, including electrical stimulation^27–38^. The fibre type ratio (FTR) between slow contracting, oxidative type I and fast contracting, glycolytic type II muscle fibres is therefore variable and adaptable. One sign of such a transformation process can be an increased number of hybrid fibres^34,36^. Electrical stimulation of the PCA, depending on the applied intensity, can change the muscle properties in the long term because it overrides the physiological recruitment pattern of the muscle fibres^38–40^. This can lead to a transformation to a slow phenotype, with a loss of maximum muscle power and contraction velocity to such an extent that it is assumed that, with a further decrease, the muscle could no longer fully relax between the artificially generated contractions. On the contrary, the fatigue resistance increased^40^. Therefore, the use of long term stimulation with specific frequencies and higher DCs may lead to muscle atrophy^40–44^. Jarvis 1993 was able to show a loss of muscle weight of up to 50% after continuous stimulation with 10 Hz and a DC of 100%. Therefore, a maximum DC of 35% is currently used in BVFP patients experimentally treated with an implanted LP for safety reasons. In addition, nerve damage and chronic inflammation, which cannot be ruled out when using long term stimulation with implanted electrodes, can also induce atrophy of muscle fibres and fibrosis^45^, which is characterised by an increased amount of intramuscular collagen ^46–49^. However, FES has also been shown to prevent atrophy of denervated and reinnervating muscles^34,49–56^, and to have a protective effect against fibrosis ^42,49,57^.

As already mentioned, patients with BVFP would benefit most from a long opening of the glottis thus high DCs for sufficient inspiration. With the present study, we aim to verify whether long-term FES with high DCs of 40% and 70% in combination with stimulation parameter, which has already turned out effective for opening, leads to critical changes in the PCA that diminish the muscle function to such a degree that the use of high DCs must be dissuaded. For this purpose, we utilised a sheep animal model because the sheep larynx is comparable to the human larynx in size and anatomy^20,58^. The changes in FTR, fibre size, the percentage of hybrid fibres, and the collagen amount of the PCAs were quantified by automated analysis of fluorescence microscopy images and compared for animals that received different long-term FES treatments.

## Methods

### Animal Care

The study was performed in accordance with the European and German animal welfare regulations. All experiments were approved by the Committee for Animal Research of the State of Thuringia, Germany (animal experimental code: UKJ-17-051). Husbandry and all medical treatments took place at the “Animal Facility and Services” of the University Hospital Jena. All authors confirm that the study complied with the ARRIVE guidelines.

The number of sheep used in this study was defined in adherence to the principles of the 3R framework, which encourages *Replacement*, *Reduction*, and *Refinement* in animal research. Furthermore, our objective was to employ a human-like animal model to ensure the direct applicability of our findings in clinical contexts. Consequently, the animal number was carefully considered to ensure optimal animal welfare, appropriate handling and availability. Instead of employing an excessive number of animals, we maximised the utility of the data by acquiring multiple image samples per animal and applying appropriate statistical methods that account for potential dependencies between data points, while still utilising all samples from each animal. Additionally, we decided to report not only measures of significance but also effect sizes that provide a more comprehensive understanding of group differences especially in case of small sample sizes.

### Animals and study design

In total, the study included 34 healthy, non-pregnant, female Merino sheep aged between four and five years. The weight ranged from 70 to 100 kg. Before enrolment, the health status of each animal was verified by a veterinarian. The attribution of animals to the distinct experimental groups was performed randomly. Twelve animals had to be excluded during the implementation of the study due to electrode disruptions (n = 10), electrode dislocation (n = 1), or complications during wound healing (n = 1). We decided against revision surgeries, as each one could influence the results of the histological examinations and obscure the impact of the electrical stimulation.

The resulting animal numbers per experimental group are outlined in Table 1. The left PCAs of all animals were temporarily paralysed by cryo-damaging the left RLNs as described in the section *Surgical and postoperative procedures* below. Cryo-damage results in axonotmesis in which only the axon is damaged, so good reinnervation can be expected. Therefore, our model is somewhat different from the clinical picture of BVFP, as most patients are more likely to have neurotmesis, which represents more severe damage to the RLN and in which reinnervation is often worse and synkinetic. However, cryo-damage still leads to de- and reinnervation and has the advantage that the effects of electrical stimulation on the PCAs can be better assessed, as the muscles of the animals are in a similar state at the beginning of the stimulation phase.

**Table 1:** Study design and animal numbers per experimental condition.

Group	Number of animals	Cryo-damage (left RLN)	Electrode implantation	Stimulation (FES)	Duty cycle (DC) (%)	Burst frequency (Hz)	ON (s)	OFF (s)	Pulses per day	Daily frequency (Hz)
CT	4	+	−	−	–	–	–	–	–	–
SHAM	8	+	+	−	–	–	–	–	–	–
DC04	4	+	+	+	40	30	1.7	2.6	1,024,740	12
DC07	6	+	+	+	70	30	3.0	1.3	1,808,372	21

*CT* control, *DC* duty cycle, *FES* functional electrical stimulation.

In addition to the nerve damage, a bipolar spiral tip electrode was implanted in the left and right PCA of 18 sheep and connected to the implantable pulse generator (IPG; for use in animals only) placed subcutaneously on the neck. Eight animals of these 18 sheep served as SHAM group and received no electrical stimulation, while 10 animals were assigned to long-term FES at 30 Hz with DCs of 40% (DC04, n = 4) or 70% (DC07, n = 6). All animals were healthy at the end of the study.

### Surgical and postoperative procedures

**Figure 1:**
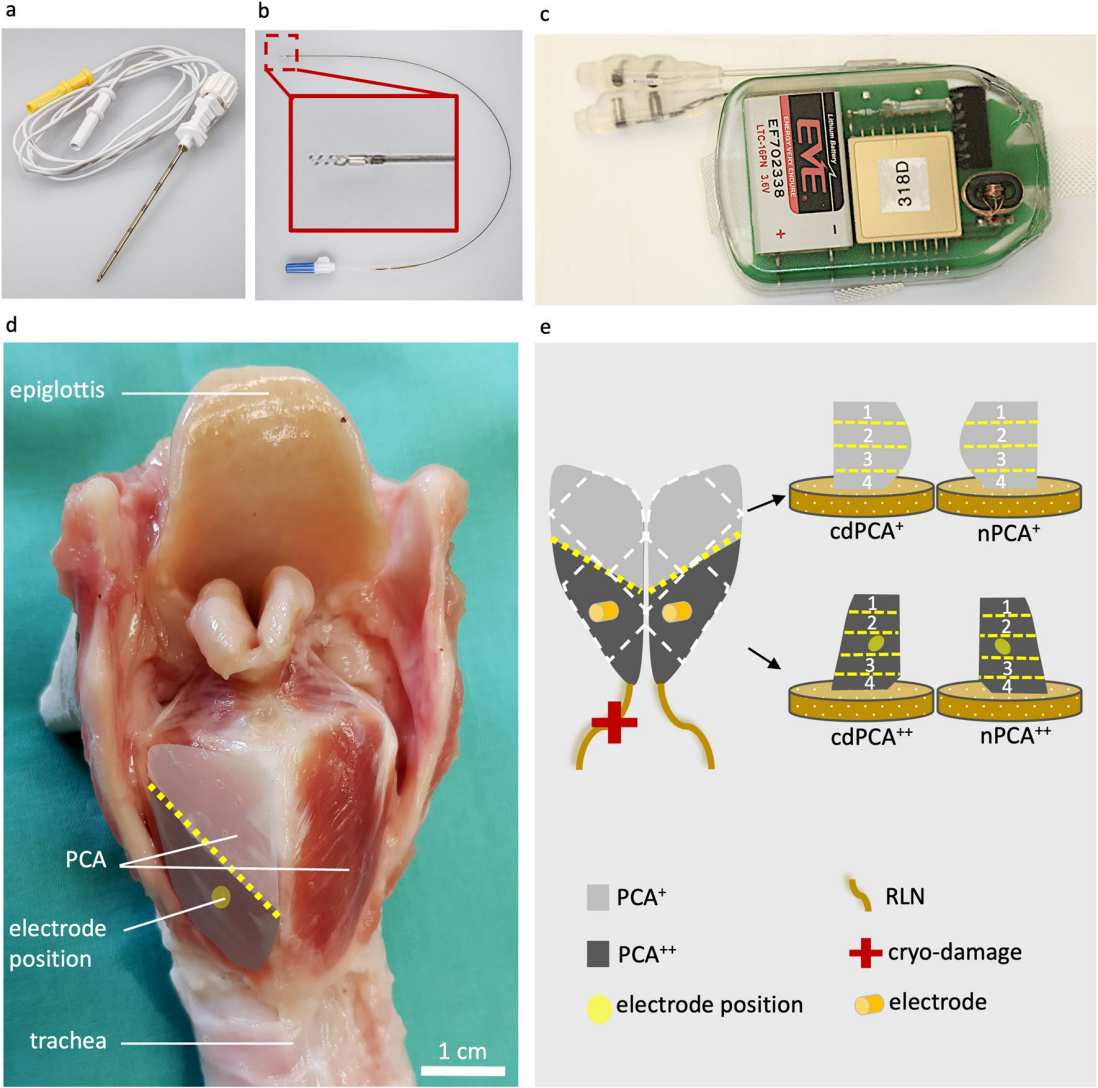
Implantable pulse generator, excised larynx with PCA, and schematic representation of the PCA. The IPG electrode implantation utilised (**a**) an insertion tool (Pajunk, Geisingen, Germany). The IPG itself consisted of (**b**) two bipolar, spiral tip electrodes (enlarged in the dark red box, length of the spiral tip = 2.1 mm, 1 mm inter distance and of the ring = 1.5 mm) and (**c**) the control unit [(**a–c**) courtesy of MED-EL, Innsbruck, Austria]. (**d**) The larynx (posterior view) of each sheep was excised to dissect the RLNs and the PCAs as shown in (**d,e**). The light grey area in (**d**) symbolises the PCA^+^ region, i.e. farther away from the electrode, and the dark grey area the PCA^++^ region, i.e. close proximity to the electrode. The approximate cutting lines for the preparation of the left (cryo-damaged RLN, cdPCA) and right PCAs (natural RLN, nPCA) into distinct pieces are shown as yellow dashed lines. The electrodes are highlighted as orange cylinders and the former positions of the electrodes as pale-yellow circles. The white dashed lines correspond to the partitioning of the PCAs into imaging samples 1 to 4, respectively.

All surgical procedures were performed under sterile conditions and general anaesthesia without muscle relaxants. The animals lay in the supine position during intubation and the implantation of the electrodes into the PCA was performed as described by Förster et al. 2013^2^. In brief, the cricoid cartilage and the upper part of the trachea were exposed by a longitudinal skin incision. A temporary tracheostoma was performed to allow an unobstructed view of the larynx and the vocal folds because ventilation via a laryngeal mask turned out to be difficult in sheep. One bipolar spiral tip electrode (3 French, MED-EL, Innsbruck, Austria; Fig. 1b) of the IPG (Fig. 1c) was placed transversally into the PCA of each side using an insertion tool (Pajunk, Geisingen, Germany; Fig. 1a) via the cricothyroid membrane, the subglottic soft tissue space, and the cricoid lamina. It was sutured to the cricoid cartilage and the placement was confirmed by electrical stimulation under visual monitoring via laryngoscopy.

An unilateral VFP was mimicked by artificially damaging the RLN (axonotmesis) as described by Cercone et al. 2019^59^. Approximately 2 cm of the RLN were exposed lateral to the trachea and close to the caudal edge of the PCA and cryo-damaged with a previously sterilised copper screwdriver precooled in liquid nitrogen. The success of the axonotmesis was verified by stimulating the nerve proximal and distal to the damaged area.

The IPG (Fig. 1c) was implanted in the left neck after repositioning the animal on its side, fixed to the musculature with a mesh by non-absorbable suture (Polyester 2/0 non-absorbable, Catgut GmbH, Germany) and connected to the electrodes. In contrast to the implants planned for human use, the IPG cannot be charged inductively, instead it contains a battery that provides the required current for the entire stimulation period of six months. Finally, the IPG function was tested under laryngoscopic control and the stimulation thresholds and currents for optimal (maximal) glottal opening were determined. After re-intubation, the tracheostoma was closed, as described in Förster et al. 2013^2^. Sterile dressing with Inadine (M PVP IOD 9.5 × 9.5 cm pads, Systagenix Wound Management Ltd, UK), Artiflex soft (BSN medical GmbH, Germany) and an elastic bandage (Elastomull haft hospital, Leukoplast, Germany) was applied during the wake-up phase. Postoperatively, the animals received analgesic and antibiotic treatments as described in detail in Additional File 1: Information S1.

A healing period of six months followed to enable reinnervation of the cryo-damaged RLN. Before the stimulation phase of an additional six months, the position of the electrodes and their condition were checked by X-ray under sedation (ketamine hydrochloride 15 mg/kg, midazolam hydrochloride 0.2 mg/kg, and propofol 0.2–0.3 mg/kg), and the stimulation parameters for maximal opening of the vocal folds were determined under endoscopy through the nose. This procedure was repeated one year after the implantation during a final control at the end of the stimulation period. All sheep were euthanised under deep anaesthesia by overdose of pentobarbital-sodium with 100—150 mg/kg (Euthadorm 500 mg/ml, CP-Pharma, Germany).

The larynxes of the animals were excised post-mortem and both the RLNs and PCAs were dissected and prepared for the subsequent analyses (Fig. 1d, e).

### Functional electrical stimulation

The FES + group comprised n = 10 animals, which received long-term FES 24 h per day with DC04 (n = 4) or DC07 (n = 6) for six months. Bipolar, rectangular pulses without a ramp were used, and it was stimulated current controlled with a frequency of 30 Hz, a pulse duration of 0.5 ms, an amplitude range between 0.7 and 2.8 mA. The left side of three animals required higher amplitudes of 3.5 mA, 5 mA and 6 mA for a maximal glottal opening. In DC04, each stimulation lasted for 1.7 s followed by a pause of 2.6 s. In DC07 the stimulation lasted for 3.0 s, followed by a pause of 1.3 s. Ultrasound was used once a week to observe the movement of the anterior parts of the vocal folds and the arytenoid cartilages^60,61^ to confirm that the PCA was still opening the vocal folds. If this was not the case, the amplitude was increased to achieve sufficient movement and, if necessary, the position and condition of the electrodes were checked by X-ray in the awake, standing animal.

### Nerve histomorphometry

**Figure 2:**
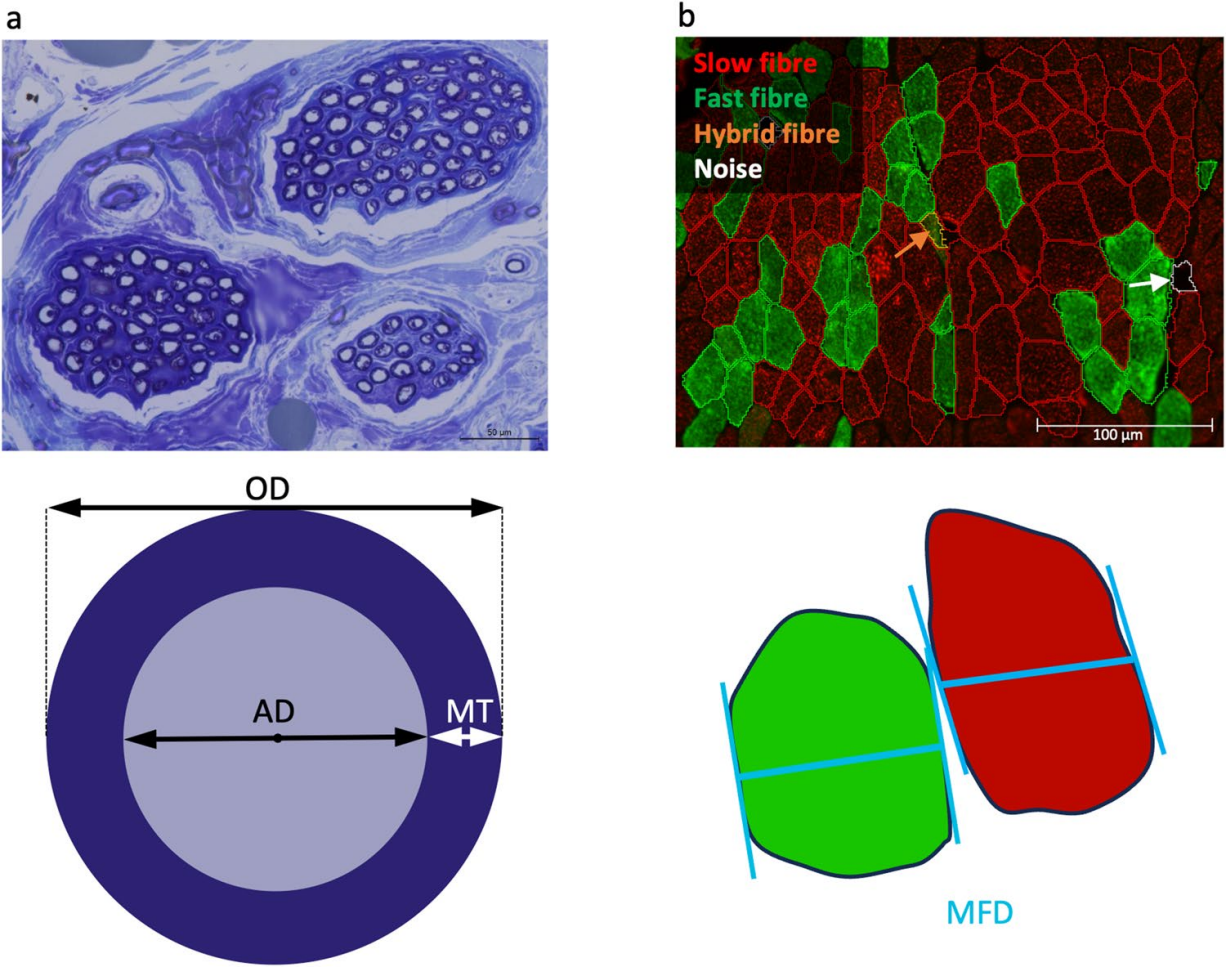
Nerve and muscle histomorphometry. (**a**) The isolated nerves were stained with toluidine blue, microscopy images were taken, and the axon diameter (AD) and the overall diameter (OD) were measured automatically with the software Volocity v6.1.1. Based on AD and OD, the myelin thickness (MT) was calculated. (**b**) Muscle cross-sections were stained for slow and fast fibres. The fibres were segmented and classified into slow (red outline), fast (green outline) and hybrid (orange outline; example highlighted with orange arrow). Segmented objects without staining were classified as noise (white outline; example highlighted with white arrow). The minimum Feret diameter (MFD) was calculated for each fibre.

Pieces of approximately 2 cm of the left and right RLNs were isolated at the distal end, the region corresponding to the nerve damage, and fixed in 5% formaldehyde where possible (in total 16 sheep divided into 11 FES^-^ and 5 FES^+^). The nerves were embedded into epoxy resin and sectioned transversally into 0.5 µm slices before staining with toluidine blue. The slices were photographed as previously reported by Cercone et al. 2019^59^. The axon diameter, the myelin thickness and the overall diameter were determined automatically utilising the software Volocity v6.1.1 (PerkinElmer) (see Fig. 2 a).

### Muscle histomorphometry

The PCAs from both sides were carefully removed from the cartilage, cut into two pieces (see Fig. 1d, e), one of which contained the electrode, and placed on a cork plate with tissue mounting medium (Leica, Germany). The samples were frozen for 20 s in melting isopentane precooled in liquid nitrogen at −156 °C. The samples were then stored in liquid nitrogen until sectioning.

The left (with cryo-damaged RLN = cdPCA) and right (natural RLN = nPCA) PCAs were divided into two blocks (see Fig. 1d, e) and are referred to according to the status of electrode implantation: (i) PCA^-^ if there was no electrode implanted at all (only CT group), (ii) PCA^+^ for the muscle part farther away from the implantation site, and (iii) PCA^++^ for the muscle part in close proximity to the electrode implantation area (see Fig. 1e). The PCA blocks were cut into 10 µm cryo-sections with a freezing microtome (Leica CM 3050S, Leica), air dried to glass slides (HistoBond adhesive slides, Paul Marienfeld GmbH & Co. KG) and stored at −21 °C.

For analysis of muscle fibres and collagen amount, cryo-sections were stained by immunofluorescence. Therefore, three different primary antibodies (myosin slow, myosin fast and collagen), two secondary antibodies (Alexa 568 and Alexa 647) and a Zenon antibody complex (Alexa 488 Zenon) were used as described in Additional File 1: Information S2. The stained cryo-sections were then fixed and mounted under coverslips.

### Microscopy imaging and image analysis

Imaging was performed using the ZEISS Axio Imager Z1 (Zeiss Microscopy GmbH, Jena, Germany) with a plan apochromatic objective with 20× magnification. One image was captured from each slide at a random position. We verified whether the utilised field of view (FOV) of randomly selected muscle areas is representative of the fibre type composition of whole cross-sections using additional samples of the vastus lateralis muscle. To this end, we compared the FTR, i.e. the number of slow fibres divided by the number of fast fibres, of small FOV images with the corresponding full cross-sections of four animals.

After image acquisition, fibres were classified into slow, fast and hybrid fibres, and the minimum Feret diameter was measured by automated image analysis using Fiji v1.53t^62^ and Cellpose^63^ (see Fig. 2b). The workflow consisted of three main steps: (i) image pre-processing, (ii) fibre segmentation, and (iii) fibre classification, and is outlined in detail in Additional File 1: Information S3. In brief, images were first converted to 8-bit grayscale. As pre-processing, the collagen structures in the orange channel were enhanced by histogram stretching and subtracted from the sum of the green (fast fibres) and the red (slow fibres) channels. The segmentation was performed using Cellpose and the extracted labels were converted into Fiji ROIs. The image channels were binarised into foreground and background regions. All ROIs not touching the image borders were then classified as being a slow, fast or hybrid fibre, or other structures. This classification was based on the size of the ROIs and the area covered in the binarised green and red channels. The number of slow fibres was divided by the number of fast fibres, giving the FTR. Additionally, the binarised collagen channel was used to quantify the area covered by collagen for each image as a measure of the intramuscular collagen amount.

The automated classification of fibres was verified by manual annotation. We therefore randomly selected three images per experimental group, and the segmented objects were classified by an expert into fast, slow, hybrid fibre and other structures. The manual annotations were then compared with the results of the automated classification.

### Statistical analysis

All statistical tests were performed with the software R v4.1.0. For the nerve morphometrics, a t-test was performed, and statistical significance was assumed for p < 0.05. For each muscle parameter, p-values across experimental conditions were calculated using a linear mixed model with the animal being a random factor, as the analysis included multiple images per sheep. The Satterthwaite's method^64^ was used to determine the degrees of freedom. The Benjamini–Hochberg correction was applied to correct for multiple testing and to calculate adjusted p-values (q-values). Statistical significance was assumed for q < 0.1. In addition, pairwise effect sizes were determined by calculating Cohen's D (d), where d is interpreted as a small effect for values around d = 0.2, as a medium effect for values around d = 0.5 and as a large effect for values around d = 0.8 and larger. For data presented as boxplot, the box extends from the first to the third quartile (Q1 and Q3), i.e. the interquartile range (IQR), the horizontal line represents the median, and the white dot represents the mean. The end points of the whiskers are calculated as Q1/Q3 ± 1.5 ∙ IQR. Black dots outside the whiskers represent outliers.

## Results

### Cryo-damage of the RLN does not lead to macroscopic changes of PCA muscles

The spontaneous movement of the vocal folds was still clearly restricted on the cryo-damaged side at the beginning of the stimulation period. At each weekly ultrasound control during the following six months under stimulation, proper movements of the vocal folds and arytenoid cartilages were always detectable in all sheep of the DC groups. At the final laryngoscopic inspection, all vocal folds moved maximally under stimulation and properly without, because the sedation allowed nearly normal breathing. Both PCA sides appeared identical in colour in the macroscopic assessment, and had a size of approximately 3 × 1.5 cm. There was visually no difference in muscle thickness.

### Cryo-damage but not electrical stimulation causes reduction of RLN diameters

**Figure 3:**
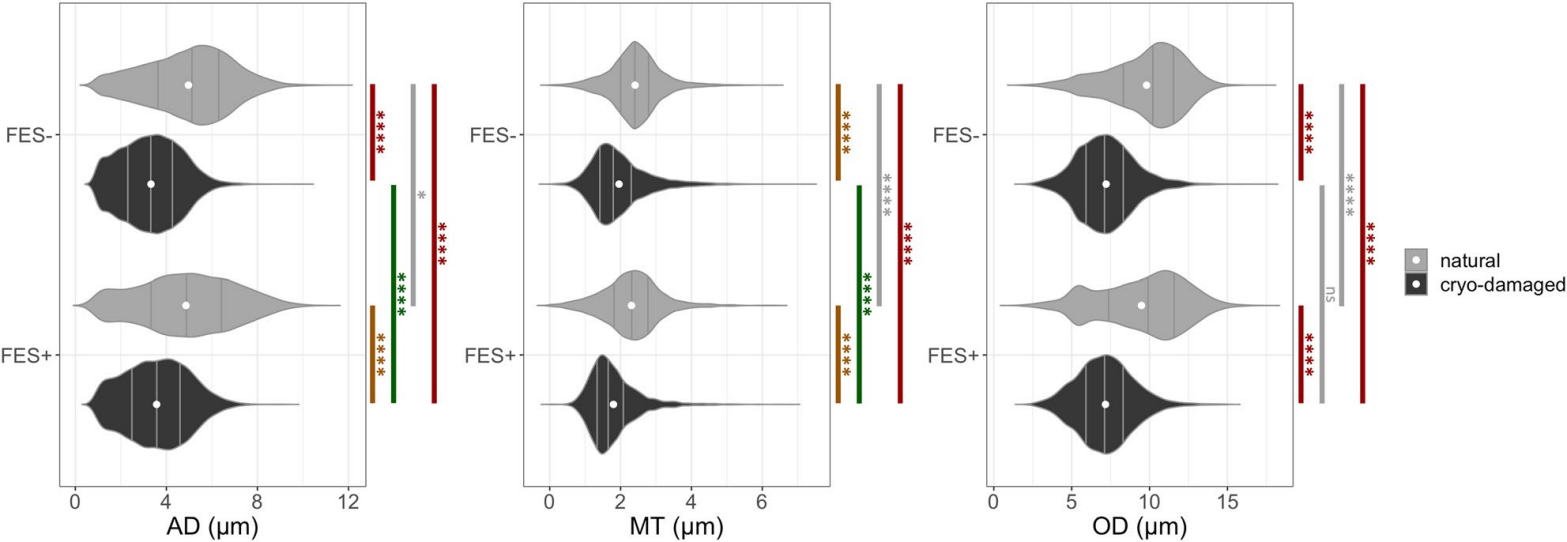
Nerve morphometrics of cryo-damaged and natural RLNs with and without FES treatment. Axon diameter (AD), myelin thickness (MT) and overall diameter (OD) showed significant differences between the cryo-damaged and natural RLNs in both FES groups (p < 2.20 × 10^–16^ each, Cohen’s D ranging from d = 0.60 to d = 1.46). The effects were less pronounced comparing cryo-damaged and natural RLNs regarding FES^-^ vs. FES^+^, respectively (p ranging from p = 0.24 to p = 2.20 × 10^–16^ and Cohen’s D ranging from d = 0.02 to d = 0.21). White dots denote mean values and gray lines denote quartiles. Labeling correspond to: ns = p-value > 0.05, *p-value ≤ 0.05, **p-value < 0.01, ***p-value < 0.001, ****p-value < 0.0001; gray: d < 0.2 no effect, green: d ≥ 0.2 medium effect, yellow: d ≥ 0.5 and red: d ≥ 0.8. See Additional File 1: Fig. S4–S6 for detailed statistics of all group comparisons.

The quantitative analysis of the RLNs (see Fig. 3 and Additional File 1: Figs. S4–S6) revealed significant differences in axon diameter, myelin thickness and overall diameter between the cryo-damaged and natural RLNs (p < 1∙10^–16^ each) with medium to large effects ranging from d = 0.68 to d = 1.6 for FES^-^ and FES^+^. The mean axon diameter, myelin thickness and overall diameter of the cryo-damaged RLNs were significantly smaller than those of the natural RLNs. The axon diameter was 36–51% thicker, the myelin thickness 23% and the overall diameter 32–36% in comparison to the cryo-damaged nerves. In contrast, the overall diameter of the axons, the axon diameter themselves and the myelin thickness of the RLNs of the FES^-^ and FES^+^ groups showed only negligible to small effect sizes ranging from d = 0.02 to d = 0.21 (p-values ranging from p = 0.24 to p < 1∙10^–16^). The area of the myelinated axons was 75% bigger in the natural RLNs than in the cryo-damaged RLNs.

### Fibre type ratio is predominantly influenced by electrical stimulation

**Figure 4:**
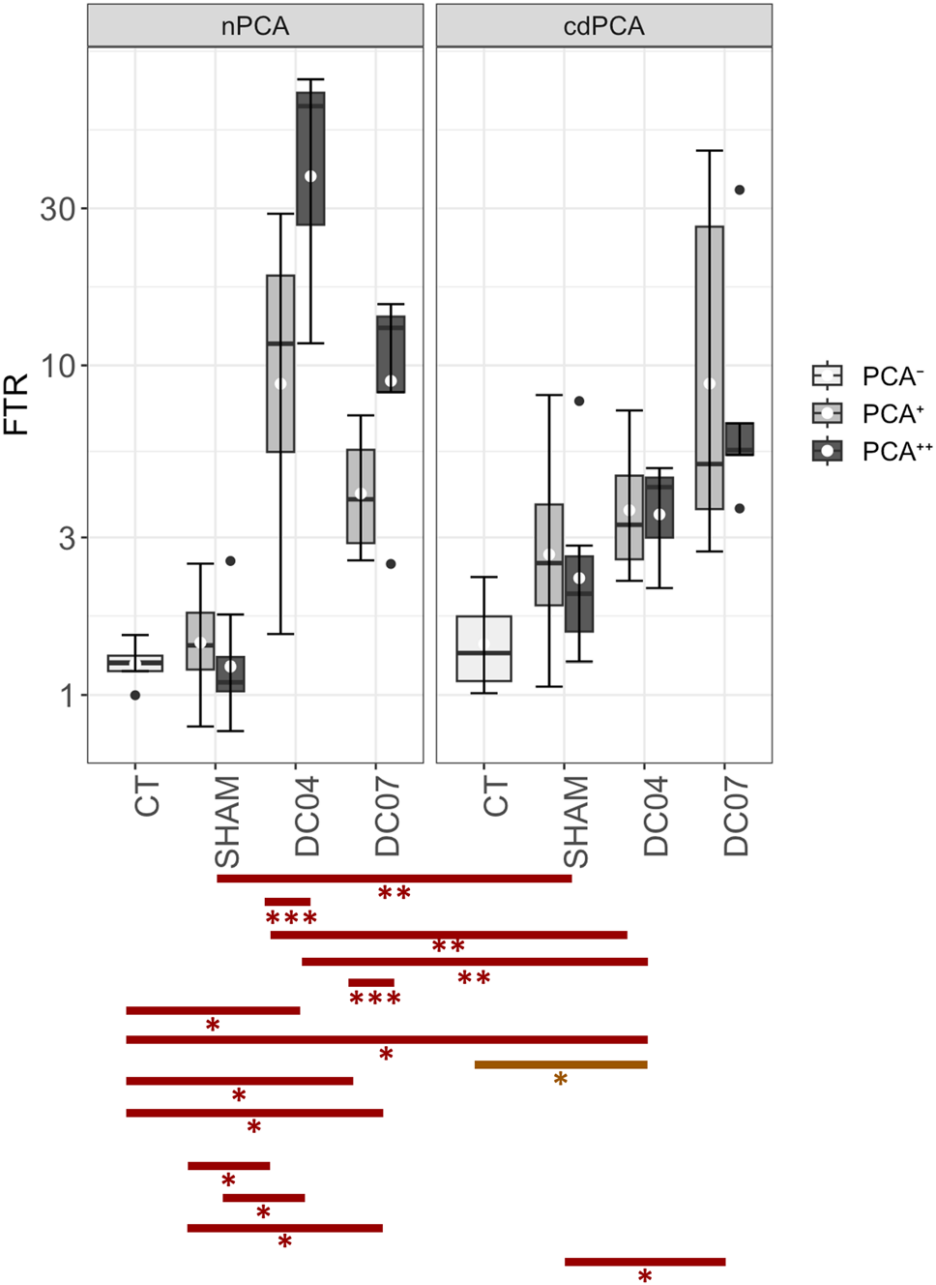
Fibre type ratio (FTR). The FTR of the CT group showed slightly elevated FTR values for cdPCAs compared to nPCAs (q = 0.14; d = 0.57). The mean FTR values of both nPCAs and cdPCAs of SHAM were always higher than those of CT. The most prominent differences in FTR were found across the FES status, independent of the RLN damage. All mean FTR values of the FES^+^ group were higher than those of the FES^-^ group (mean_FES−_ < 3.21 and mean_FES+_  > 3.75) regardless of the duty cycle. The y-axis is plotted as logarithmic values. White dots denote mean values and the black lines denote the median. Labeling corresponds to: *q-value ≤ 0.1, **q-value < 0.01, ***q-value < 0.001, ****q-value < 0.0001; gray: d < 0.2 no effect, green: d ≥ 0.2 medium effect, yellow: d ≥ 0.5 and red: d ≥ 0.8. See also Additional File 1: Fig. S7 for detailed statistics of all group comparisons.

The quantification of FTR (see Fig. 4 and Additional File 1: Fig. S7) based on small FOV images was validated by comparing the FTR values of a test dataset with the FTRs of corresponding whole muscle cross-sections. No difference was found between the small FOVs and the whole cross-sections (FTR_small_FOV_ = 0.40 ± 0.16, FTR_cross-section_ = 0.44 ± 0.15; p = 0.74). Additionally, the automated image analysis was validated by comparing the classification results with manual annotations of randomly selected microscopy images. It was ensured that each experimental group was represented in the validation dataset. On average, 95,62% of the fibres in an image were classified correctly by the automated analysis (median = 97.92%, IQR = 9,62%).

The mean FTRs did not significantly differ between the nPCAs and cdPCAs within the CT group (q = 0.14, d = 0.57). Likewise, there was no significant difference between CT and SHAM, although medium to large effects ranging from d = 0.66 to d = 1.03 were observed when comparing cdPCAs. The cdPCAs of the SHAM group also showed a trend towards higher FTR values compared to the nPCAs of this group (d = 0.98, q > 0.1 for cdPCA^+^ versus nPCA^+^; d = 0.89, q = 0.01 for cdPCA^++^ vs. nPCA^++^).

We observed more pronounced differences for the FTR within the groups DC04 and DC07. The nPCAs of DC04 had overall higher FTR values, especially within the nPCA^++^ region, which is reflected by a significant difference between nPCA^++^ and nPCA^+^ (q = 6 ∙ 10^–4^, d = 1.55) as well as for the nPCA^++^ versus the cdPCA^++^ (q = 1.9 ∙ 10^–3^, d = 1.94). Similarly, also the FTR of nPCA^+^ was significantly higher than for cdPCA^+^ (q = 0.03, d = 1.15). In contrast, the only significant difference found within the group DC07 was higher FTR values for nPCA^++^ compared to nPCA^+^ (q = 0.08, d = 1.51). Although not reaching statistical significance, we also observed a medium effect for the comparison of nPCA^+^ versus cdPCA^+^ in DC07 (q = 0.18, d = 0.79), with higher FTRs in cdPCA^+^. Overall, there was a tendency for an increased FTR in DC04 and DC07 in comparison to CT regardless of the PCA condition and electrode position (d > 0.97 each). Additionally, a significantly higher FTR in the nPCAs of both FES^+^ groups, regardless of electrode position was found (q < 0.2 and d > 1.10, each), as well as a tendency in the cdPCA regions towards an increased FTR compared to the SHAM group (q > 0.10, each; Cohen´s D ranging from d = 0.37 to d = 0.81).

In general, all mean FTR values of the FES^+^ group were larger than those of the FES^-^ group, independent of the duty cycle. Additionally, the mean FTR values of the PCAs of the SHAM group were always higher than those of the CT group.

### Muscle fibre diameter is not substantially affected by RLN damage or electrical stimulation

**Figure 5:**
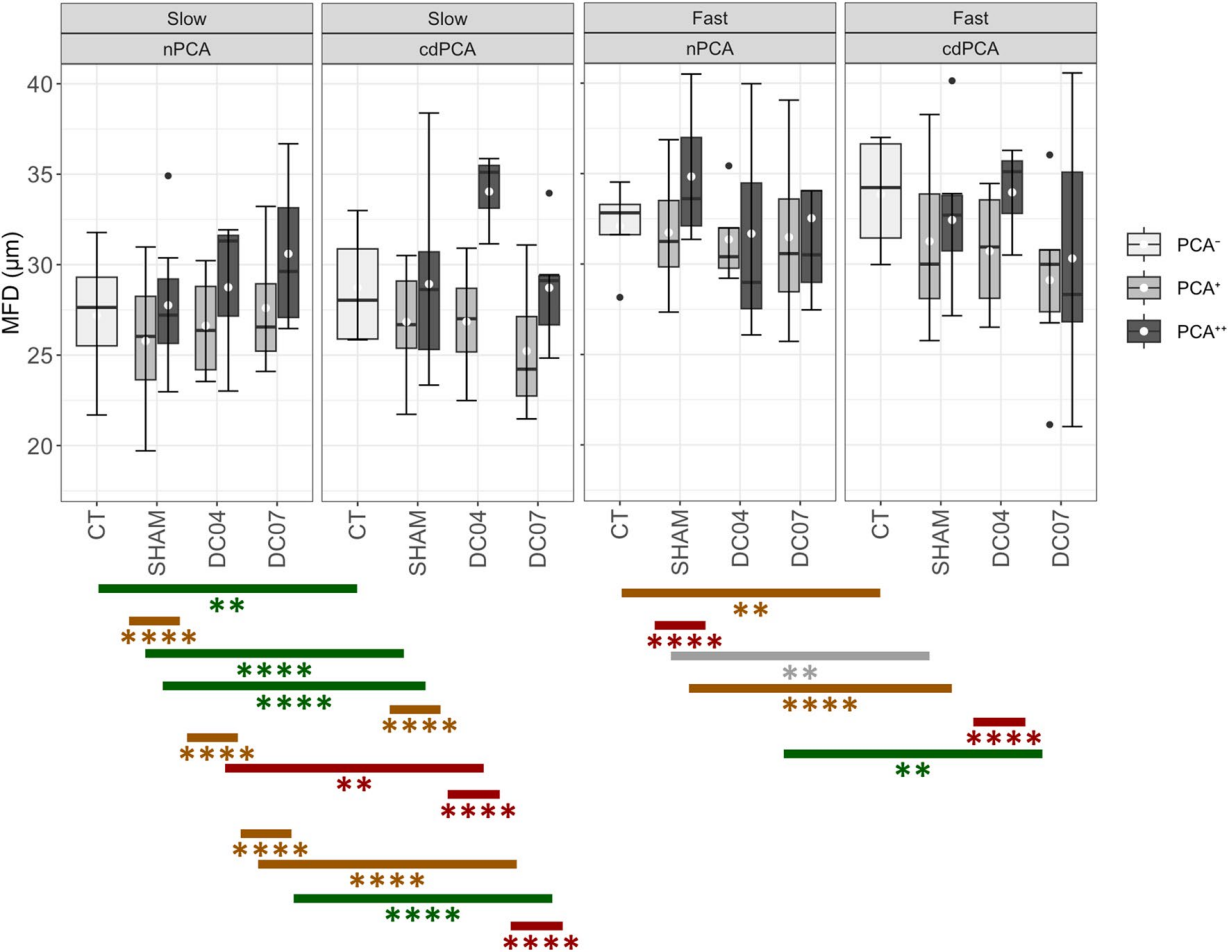
Fibre diameter of slow and fast fibres. The fibre diameter corresponds to the minimum Feret diameter (MFD) of the fibres. The mean MFDs of slow fibres tended to be lower than the mean MFDs of fast fibres except for DC04 cdPCA^++^. Both slow and fast fibres showed no significant differences across groups, but only within groups. For the slow fibres of both stimulation groups, i.e. DC04 and DC07, PCA++ regions showed an increase in fibre diameter independent of nerve damage. For fast fibres, significant differences with large effects were found for SHAM nPCA^++^ vs. SHAM nPCA^+^ and DC04 cdPCA^++^ vs. DC04 cdPCA^+^. White dots denote mean values, and the black lines denote the median. Labeling correspond to: *q value ≤ 0.1, **q-value < 0.01, ***q value < 0.001, ****q-value < 0.0001; gray: d < 0.2 no effect, green: d ≥ 0.2 medium effect, yellow: d ≥ 0.5 and red: d ≥ 0.8. See also Additional File 1: Figs. S8 and S9 for detailed statistics of all group comparisons.

The fibre diameter was measured as the minimum Feret diameter and is shown in Fig. 5 for slow and fast fibres, respectively. All statistical values are summarised in Additional File 1: Figures S8 and S9. In general, all changes in fibre diameter were below 8 µm, and significant differences in the fibre diameters for slow and fast fibres appeared exclusively within groups. Slow fibres showed an overall smaller fibre diameter than fast fibres, except for cdPCA^++^ of the group DC04. Within CT, slow and fast fibres were significantly thicker in the cdPCAs compared to the nPCAs (q < 0.007 and d > 0.40, each). This was also true for the slow fibres of all PCA regions of the groups SHAM and DC04 (q < 10^–4^, d > 0.27, each).

For the SHAM group, we found that the fibre diameter of fast fibres of the cdPCA was significantly smaller than in the related nPCA regions, independent of the electrode position (PCA^+^: q = 0.002, d = 0.12 and PCA^++^: q = 10^–5^, d = 0.62). Additionally, the fibre diameter of fast fibres was significantly larger in the nPCA^++^ than in the nPCA^+^ for SHAM (q <  < 10^–14^, d = 0.88). Likewise, the cdPCA^++^ had thicker fast fibres than the nPCA^++^ in DC04 (q <  < 10^–5^, d = 0.93). In contrast, slow and fast fibres showed a tendency towards smaller fibres in the cdPCAs compared to nPCAs in the group DC07. While for the slow fibres this difference was significant for both nPCA regions (PCA^+^: q = 10^–14^, d = 0.63 and PCA^++^: q = 10^–4^, d = 0.49), this was only the case for nPCA^+^ and cdPCA^+^ for fast fibres (q = 0.01, d = 0.18). There was no significant difference between the nPCA of the CT group and all other experimental groups. Overall, the fibres tended to be thicker in close proximity to the electrode.

### Number of hybrid fibres increases in response to RLN damage and close to electrode position

**Figure 6:**
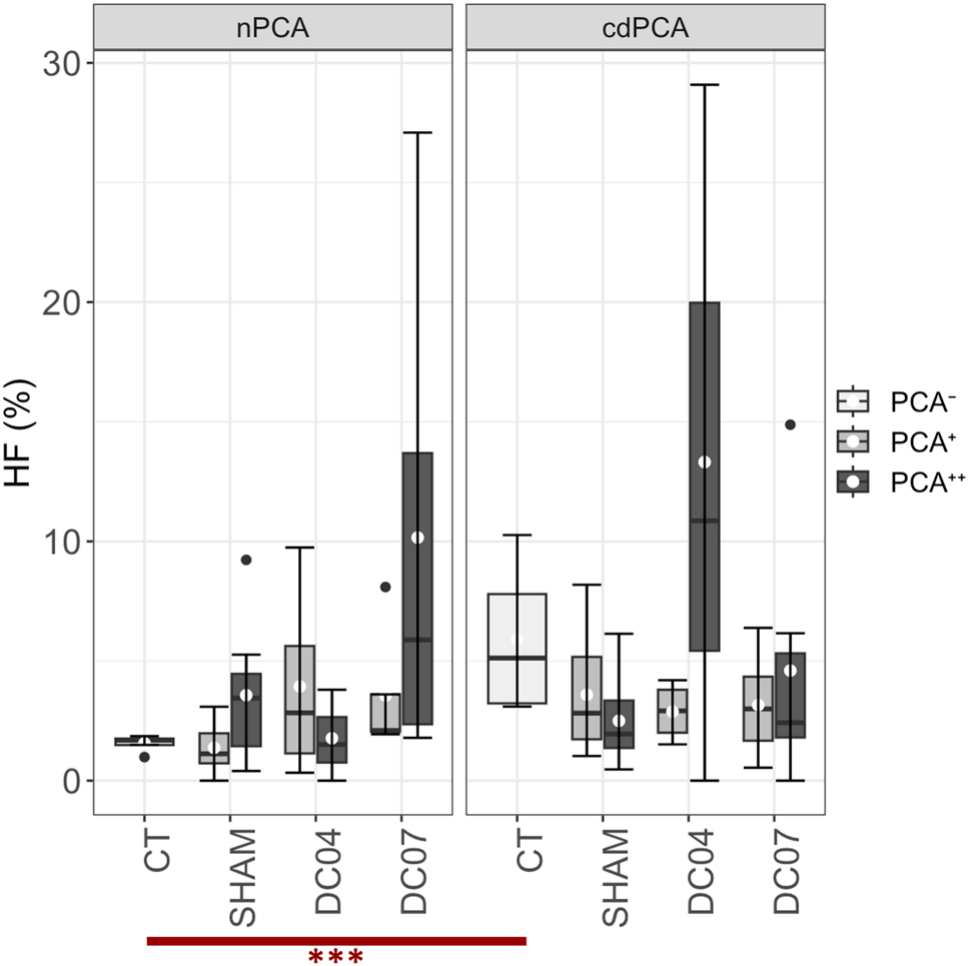
Percentage of hybrid fibres (HF). The proportion of hybrid fibres was mostly similar across groups. Only cdPCAs of the CT group showed a significant increase compared to the corresponding nPCAs. Although not yielding statistical significance, the nPCA^++^ of the group DC07 as well as the cdPCA^++^ of the group DC04 showed a high HF proportion. White dots denote mean values, and the black lines denote the median. Labeling correspond to: ***q-value < 0.001, red: d ≥ 0.8. See also Additional File 1: Fig. S10 for detailed statistics of all group comparisons.

The proportion of hybrid fibres (see Fig. 6 and Additional File 1: Fig. S10) was significantly higher for cdPCAs compared to nPCAs of CT (q = 0.002, d = 1.79). The hybrid fibre proportion also varied within SHAM with a tendency towards more hybrid fibres in nPCA^++^ compared to nPCA^+^ (q = 0.34, d = 0.99). A similar trend was found between cdPCA^+^ and nPCA^+^ in SHAM (q = 0.17, d = 1.14). A tendency towards more frequent hybrid fibres was detected in nPCA^++^ of DC07 and the cdPCA^++^ of DC04 compared to the related PCA regions of CT and SHAM (q > 0.1 and d > 0.77, each). Despite large effect sizes within and across groups, only the comparison of nPCA and cdPCA of the CT group yielded statistical significance.

### Neither RLN damage nor electrical stimulation induce clear signs of fibrosis

Each implanted electrode was surrounded by a scar of connective tissue with a thickness of approximately 500 µm, regardless of whether it was used for stimulation or not.

**Figure 7:**
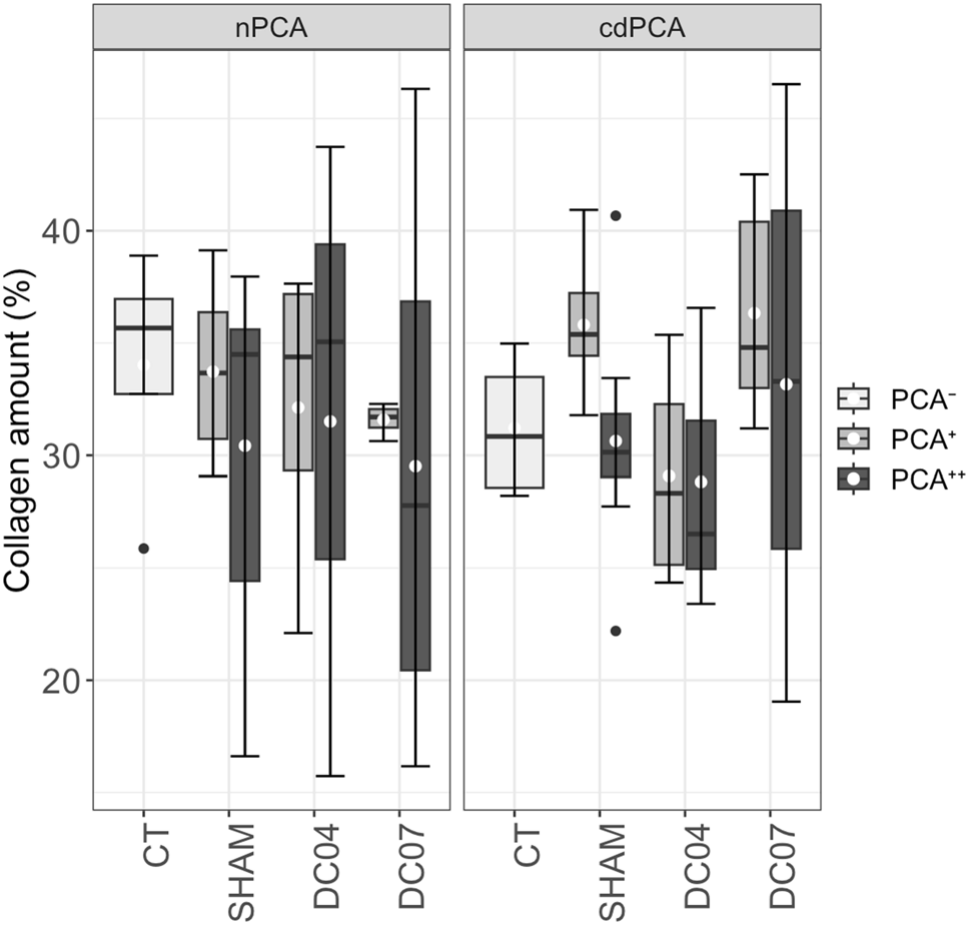
Intramuscular collagen amount. The collagen amount did not differ significantly across the groups. The mean collagen amount of the nPCA in the CT group (mean = 34.0) was higher than in the cdPCA of CT (mean = 31.2). The highest levels of intramuscular collagen were observed for cdPCA^+^ of the group SHAM and the group DC07 (SHAM cdPCA^+^: mean = 35.8; DC07 cdPCA^+^: mean = 36.3). White dots denote mean values, and the black lines denote the median. No significant differences were detected. See also Additional File 1: Fig. S11 for detailed statistics of all group comparisons.

We observed trends towards an increased amount of intramuscular collagen for cdPCA^+^ of the groups SHAM and DC07 (Fig. 7 and Additional File 1: Fig. S11). These groups yielded high effect sizes when compared to the corresponding PCA regions of CT and DC04. However, these differences were statistically not significant and therefore not assumed to be signs of clear fibrosis.

## Discussion

The purpose of this study was to quantify the effects of long-term functional electrical stimulation (FES) with high DCs on the PCAs in a sheep model in order to examine the potential effect of this treatment on the *posterior cricoarytenoid* (PCA) muscles in patients with bilateral vocal fold paresis (BVFP). Also, if it does not completely reflect the clinical picture, the recurrent laryngeal nerve (RLN) was cryo-damaged on one side in all experimental groups in order to establish conditions close to those patients. The stimulation was commenced after six months to allow a reinnervation of the PCAs. In most of the patients reinnervation also occurs, often in combination with synkinesis^65–67^.

In this study, only left RLNs were cryo-damaged while the right sides served as controls. Similar to the observations of Cercone et al. 2019 and Chao et al. 2020^59,68^, we observed the axons of the cryo-damaged RLNs to be significantly smaller in diameter compared to the natural ones. Natural side-dependent variation in RLN length and thickness has previously been reported by Shin 1971, López-Plana et al*.* 1993 and Jotz et al*.* 2011^69–71^, although these differences were not consistently associated with a particular side of the RLN. Furthermore, in this study we observed 32% to 36% larger overall diameters, with 36–51% thicker axon diameters and 23% thicker myelin sheaths for the natural side. This corresponds to a computed cross-sectional area that is approximately 75% to 86% larger in size. These differences clearly exceed the natural variation of 21% that was reported earlier for the cross-sectional area^69–71^; thus, we can conclude that axonotmesis in our experiments was successful. Nevertheless, it is astonishing that there were no visible functional limitations of the PCAs on the damaged side despite such a large reduction of the cross-sectional area. Maybe it was impossible for us to recognise existing differences because we did not measure any forces, nerve conduction velocities, myoelectrical activities or electromechanical delays. We were therefore unable to assess the functionality of the PCAs in detail. In addition, we did not observe an effect of the electrical stimulation on the RLNs’ axon diameter, myelin thickness or overall diameter.

Macroscopically, the excised PCAs with cryo-damaged and natural RLNs did not differ from each other after 6 months of stimulation. A more detailed microscopic examination was performed on fluorescently stained muscle fibres utilising automated image analysis. The effect of long-term FES with different stimulation parameters on muscle architecture was investigated in terms of fibre type ratio (FTR), fibre diameter, number of hybrid fibres, and intramuscular collagen amount.

The control group (CT) and SHAM group showed a trend towards higher FTRs in the cryo-damaged PCAs (cdPCAs) than in the natural PCAs (nPCAs), indicating that the cryo-damage of the RLN and reinnervation of the PCA muscle caused a proportional increase of slow fibres over fast fibres. This observation is consistent with earlier findings for denervated, as well as reinnervated muscles^29,30,72,73^. The effect was even more pronounced in SHAM, which might be attributed to a summation of the effects of the cryo-damage and electrode implantation. Although we observed a relative increase for cdPCAs, it has to be noted that the absolute FTR values were still close to the physiological state of the PCA^73–76^, and that the muscle can therefore be assumed not to be impaired in its contraction strength. Additionally, the electrode implantation itself did not cause an increase of the FTR as is reflected by only minor differences between the nPCAs of CT and SHAM.

A comprehensive transformation into a slow-twitch-muscle can be associated with a substantial loss of power production of up to 90% and a reduction of the contraction velocity that changes the functionality of the muscle^40,42,77,78^. We observed a clear fast-to-slow shift in the FTRs of stimulated PCAs when compared with CT, but no complete transformation. However, in contrast to Jarvis^40^ we used duty cycles of 40% (DC04) and 70% (DC07) instead of continuous stimulation, thus allowing a sufficient blood flow, oxygen supply, and metabolite removal. Furthermore, the conversion from fast to slow fibres has also been associated with increased fatigue resistance^79^. Various studies revealed that improved fatigue resistance occurs already within two weeks of chronic electrical stimulation^39,40,77,80^. This effect could be advantageous for the use of the LP in patients with BVFP, especially since these patients are stimulated for a long time.

The highest FTR, which corresponds to the highest proportion of slow fibres, was found in nPCAs of DC04. This might be attributed to the daily frequency equivalent of 12 Hz, since it is known that chronically stimulating muscles at 10 Hz causes a substantial fast-to-slow shift, up to a complete conversion into slow muscle fibres^39,40,80^. The occurrence of a fibre type shift depends on the dose of electrical stimulation^79^ and can also happen for burst stimulation^39,49,77,80^. In this study, we used a burst rate of 30 Hz, resulting in a daily frequency equivalent of 12 Hz and 21 Hz for the groups DC04 and DC07, respectively. This choice was motivated by the potential future application in humans, where the stimulation pattern can only be varied to a certain extent, because patients with BVFP require their vocal folds to be opened as wide as possible to facilitate sufficient inspiration. It should be noted that although FTRs increased in the stimulation groups, no complete transformation into a slow muscle was induced by long-term FES, and that the vocal folds were moving properly until the end of the experiment.

We can only speculate as to the reasons for the much higher FTRs in nPCAs compared to cdPCAs in the group DC04, and vice versa in the group DC07. It is conceivable that the nPCAs respond more effectively to the stimulation patterns due to their natural innervation, leading to an increased fast-to-slow shift for the stimulation at 12 Hz in the DC04 group compared to the stimulation with 21 Hz in the DC07 group. We also found a higher proportion of slow fibres in close proximity to the electrodes, although our stimulation pulses with a duration of 0.5 ms are not able to activate muscle fibres directly, but rather through the intramuscular axons. Since the electrodes were placed medio-caudally to the main nerve trunk, all motor units should always be activated simultaneously. Differences in FTR close and far from the electrode could therefore only occur if the stimulation does not reach all axons connected to muscle fibres far away due to small variations in the electrode position.

Changes in the fibre diameter provide an additional indication of the muscle condition. We found a significant difference of the fibre diameter in response to the nerve damage in the CT group. A similar effect was observed within close proximity to the electrodes for slow fibres, which could be attributed to hypertrophy of the remaining muscle fibres to compensate for fibres lost during electrode implantation. Cheetham et *al*. 2015 reported a muscle fibre atrophy by a decrease of the fibre diameter of 37% in the PCAs of horse after denervation^49^, whereas we observed only a minor decrease of less than 10% across all experimental groups compared to CT, and a maximum increase of less than 25%. These differences in the range of only a few micrometres in combination with vocal fold movement that was confirmed by video-endoscopy at the study endpoint, allows us to conclude that the contractility of the muscle was not critically affected.

Hybrid fibres possess the properties of both slow and fast fibres and are therefore an indication of an active conversion process of the muscle. While denervation and muscle atrophy can be characterised by an increased number of hybrid fibres, the stimulation of a denervated muscle triggers their differentiation into slow or fast fibres, thus causing a decrease of hybrid fibres^49^. Although the conversion process should be completed after 1 year of regeneration in our study design, we still found significantly more hybrid fibres in the cdPCA of CT than in the corresponding nPCA group. Likewise, the mean proportion of hybrid fibres was increased close to the electrode of cdPCAs in DC04 and nPCAs in DC07. Since we also observed changes in the FTR in response to RLN damage and FES, but the fibre diameter did not indicate current atrophy, the increased occurrence of hybrid fibres may be an indication of ongoing changes in muscle architecture.

We found localised fibrosis in the form of a scar of approximately 500 µm in the immediate vicinity of the electrodes in each PCA. When muscle fibres are injured, e.g. by the implantation of a foreign object such as an electrode, by long-term FES or by denervation, they can become atrophic and removed. An acute inflammatory response is then important to repair the muscle using satellite cells. If the problem persists, the inflammation becomes chronic, and non-functional muscle and fibrotic tissue are formed^48^, which can eventually cause the muscle to harden and lose its ability to contract^49^. Fibrosis of this extent could be ruled out in our study as the measured amount of collagen was not increased in any PCA region or group compared to CT.

One potential limitation of this study is the low number of animals used, which was additionally accompanied by high biological variation that was observed especially in the stimulated animals. The small animal number was not only attributed to technical limitations, but also to ethical reasons, and was compensated by gathering multiple image samples per animal in combination with appropriate statistical analysis. Still, these aspects must be considered when interpreting the results and planning future studies. Furthermore, only small FOVs of microscopy cross-sections were analysed to extract the muscle features. However, the reliability of this approach was validated and automated image analysis, which was itself evaluated by comparison with manual annotations, enabled the bias-free extraction of all muscle features from the available data.

## Conclusions

To our knowledge, this is the first comprehensive histological study with a focus on the effects of long-term FES (24 h for six months) with high DCs on PCA muscles using a laryngeal pacemaker designed for the treatment of patients with BVFP. Unlike previous studies, we examined the combined effects of artificial nerve damage, electrode implantation, and long-term FES with varying DCs. Successful axonotmesis and reinnervation of the PCAs could be ascertained through histological analysis of RLNs. Although significant fast-to-slow shifts in fibre type occurred following long-term FES, no complete conversion into a slow muscle was observed. Examination of other muscle parameters, such as fibre diameter, hybrid fibre proportion, and collagen amount, indicated that neither the electrode implantation nor the different stimulation parameters caused systematic atrophic or fibrotic changes in the PCAs. In all animals, the PCAs were functional and able to contract at the end of the experiment. Our findings demonstrate that the stimulation parameters and the chosen high DCs utilised in this study are suitable for BVFP treatment. Furthermore, since no substantial changes were detected concerning distinct DCs, it suggests that dynamic DCs could potentially be employed in BVFP patients to enable adaptive FES, e.g. in response to light physical stress.

### Supplementary Information

Supplementary Information.

## Data Availability

The datasets generated and analysed during the current study are available at: https://asbdata.hki-jena.de/WalluksHoffmannEtAl.

## Abbreviations

AD: Axon diameter
cdPCA: RLN damaged PCA
CT: Control
DC: Duty cycle
DC04: Stimulation group with 40% DC
DC07: Stimulation group with 70% DC
FES: Functional electrical stimulation
FTR: Fiber type ratio
HF: Hybrid fibre
MFD: Minimum ferret diameter
MT: Myelin thickness
nPCA: Natural PCA
OD: Overall diameter
PCA: Posterior cricoarytenoid muscle
PCA^−^: PCA without electrode
PCA^+^: PCA far away from electrode
PCA^++^: PCA close to electrode
RLN: Recurrent laryngeal nerve
SHAM: Placebo group with electrodes
