# Supplementary material for: Long-term stimulation by implanted pacemaker enables non-atrophic treatment of bilateral vocal fold paresis in a human-like animal model
Source: Sci Rep. 2024 May 7;14:10440. doi: 10.1038/s41598-024-60875-0 (PMC11076618; doi:10.1038/s41598-024-60875-0)
Supplement: Supplementary file 1 — Supplementary Information. [file 41598_2024_60875_MOESM1_ESM.pdf]

Supplementary Information

Long-term stimulation by implanted pacemaker enables non-atrophic treatment of bilateral vocal fold paresis in a human-like animal model

Kassandra Walluks[1,2,3,*], Bianca Hoffmann[1,*], Carl-Magnus Svensson[1], Gerhard Förster[4], Andreas H. Müller[4], Jonathan Jarvis[5], Justin Perkins[6], Marc Thilo Figge[1,7,**], Dirk Arnold[8,**]

[1] Applied Systems Biology, Leibniz Institute for Natural Product Research and Infection Biology – Hans Knöll Institute, Jena, Germany

[2] Faculty of Biological Sciences, Friedrich Schiller University Jena, Jena, Germany

[3] Institute of Zoology and Evolutionary Research, Faculty of Biological Sciences, Friedrich Schiller University Jena, Jena, Germany

[4] Clinic for Otorhinolaryngology & Plastic Surgery, Waldklinikum Gera, Gera, Germany

[5] John Moores University, Faculty of Science, Sport and Exercise Sciences, Liverpool, United Kingdom

[6] Royal Veterinary College London, London, United Kingdom

[7] Institute of Microbiology, Faculty of Biological Sciences, Friedrich Schiller University Jena, Jena, Germany

[8] Clinic and Polyclinic for Otorhinolaryngology, University Hospital Jena, Jena, Germany

* Authors contributed equally

** Authors contributed equally. Correspondence should be addressed to dirk.arnold@med.uni-jena.de, thilo.figge@leibniz-hki.de

S1 - Analgesic and antibiotic treatment

Starting from the second postoperative day, the dressing was changed daily, and the wound healing was checked. The stitches were removed after approximately ten days, and the dressing after another two days, as long as the animal did not manipulate the wounds. For analgesic coverage, animals received a maximum of 5 ml/kg of meloxicam 0.5 mg/kg/d or carprofen 4.0 mg/kg/d, and concomitant metamizole 25-50 mg/kg/12 h via an indwelling venous catheter for the first three postoperative days. In addition, animals were treated intravenously with ampicillin 10 mg/kg/12h for 7 days, and enrofloxacin 5 mg/kg/12h for additional three days. From the fifth postoperative day, 25-50 mg/kg/12h of metamizole were administered intravenously or intramuscularly for analgesia, as needed. Starting from the seventh day, if necessary, animals received 20 mg/kg of oxytetracycline 15 min after administration of 1 ml of lidocaine.

S2 - Immunofluorescent staining

The PCA sections were thawed for 30 min, followed by 15 min of fixation in a 1:1 methanol-acetone solution (Roth®) at -20°C. Before placing the slides into the slide racks (ThermoScientific®), they were washed with Phosphate buffered saline (PBS, SIGMA Aldrich®) and mounted on Coverplates™ with PBS. After a twenty-minute incubation with 100 µl of 0.5% Triton X – 100 at room temperature (RT), a washing step with three times 100 µl PBS followed. The samples were incubated for 1 h with 100 µl of an antibody solution containing the primary antibodies Collagen VI (Collagen VI - rabbit IgG Polyclonal, Abcam®, 1:1000, RRID:AB_305585) and Myosin-Slow (Myosin Slow - mouse IgG1 Monoclonal 1:250 in PBST, Abcam®, 1:250, RRID:AB_297660) at RT. Afterwards, the slides were washed three times for 5 minutes with 100 µl PBS and incubated for 1 h with 100 µl solution of the secondary antibodies Alexa anti-Rabbit 568 (Alexa Rabbit 568, 1:1000, Thermo Fischer®, RRID:AB_143157) and Alexa anti-Mouse 647 (Alexa Mouse 647, 1:500, Thermo Fischer®, RRID:AB_2534108) in PBST. After the next washing step with three times 100 µl PBS, 30 µl of the third primary antibody Myosin Fast (Myosin Fast, mouse IgG1 moloclonal, SIGMA-Aldrich®, RRID:AB_477190) was incubated with 150 µl of the labeling solution (Labeling Solution, Alexa Fluor 488 Zenon™ Mouse IgG1 Labeling Kit, Invitrogen™, RRID:AB_2736941) for 5 min and the coupling reaction was blocked for 5 min with 150 µl of the blocking reagent (Blocking Solution, Zenon™ Mouse IgG1 Labeling Kit, Invitrogen™, RRID:AB_2736941). The entire reaction mixture was dissolved in 3 ml of PBS. Each slide was then incubated at RT for 1 h with 100 µl of this antibody solution. After the incubation, washing was performed four times with 100 µl PBS. The slides were then stained with 100 µl solution of DAPI (Dapi Solution, abcam® in PBS, 1:1000) and washed again two times with 100 µl of PBS. All slides were fixed with 100 µl of 4% formaldehyde solution for 15 min and washed again with 100 µl of PBS for 5 min. All slides were mounted on cover slips (cover slips thickness 1, 24x50 mm, Carl Roth®) with antifade mounting medium (Anti-Fade Fluorescence Mounting Medium, abcam®).

S3 - Automatic image analysis

The classification of fibers was performed in three steps, which are explained below.

1. Image pre-processing

First, the images were converted to 8-bit grayscale. The contrast of the green and red channel was enhanced (saturation = 0.35, normalization enabled) and both channels were convolved with a median filter (radius = 5 pixels). The contrast of the orange channel was enhanced (saturation = 1, normalization enabled), a median filter (radius = 5 pixels) was applied and a morphological closing operation was performed (disk structuring element with a radius of three pixels). The background was subtracted from this channel with a rolling ball algorithm (radius = 30 pixels).

A second convolution with the median filter was applied.

The contrast was enhanced using Fiji's inbuilt CLAHE algorithm with default settings. Finally, the green and red channel were summed und the orange channel was subtracted to obtain distinct outlines for all fibre types.

2. Fibre segmentation

The pre-processed images were used as input for the cell segmentation software Cellpose.

Individual fibers were segmented using the following

parameter settings:

pretrained_model = cyto,

diameter = 150, flow_threshold = 1,

cellprob_threshold = 6.

The resulting masks were saved as png files and converted into FIJI ROIs.

3. Fiber classification

The original images were first converted to 8-bit grayscale and the contrast was enhanced (saturation = 0.1, normalization enabled). The orange channel was convolved with a median filter (radius = 2 pixels),

→ the background was subtracted with a rolling ball algorithm (radius = 150 pixels) and

the channel was binarised with a global threshold using the Triangle method. Foreground noise was removed with the remove outliers function (radius = 2 pixels, threshold = 50, bright outliers). The collagen amount of each image was then quantified by measuring the foreground area. The green channel was binarised using k-means clustering to split the image into three classes (number of clusters = 3, cluster centre tolerance = 0.00001, randomization seed enabled, randomization seed = 48). The resulting cluster with the highest greyscale intensity was defined as foreground region. In cases where the contrast between the clusters with the highest and the second-highest intensity was lower than the contrast between the clusters with the second-highest and lowest intensity, also the cluster with the second-highest intensity was defined to belong to the foreground region. The red channel was first smoothed by applying the BEEPS plugin (range filter = Gauss, photometric standard deviation = 30, spatial decay = 0.01, iterations = 1). The channel was then binarised with a global threshold that was defined as the mean intensity of the channel multiplied by a factor of 0.9. The outlines of all ROIs were shrunk by one pixel and the foreground area was measured for the binarised green and red channels within the ROIs. A ROI was classified as belonging to the class of:

(i) hybrid fibers, if at least 40% of the ROI area was covered by foreground in both red and green channels and the ROI area was larger than 100 μm^2,

(ii) fast fibers, if at least 40% of the ROI area was covered by foreground in the green channel and the ROI area was larger than 100 μm^2,

(iii) slow fibers, if at least 40% of the ROI area was covered by foreground in the red channel and the ROI area was larger than 100 μm^2,

(iv) other objects, if none of the above criteria were met.

ROIs at the image borders were excluded from the analysis.

S4 – Axon diameter statistics

AD			FES- natural	FES- cryo-damaged	FES+ natural	FES+ cryo-damaged
	mean		4.99	3.30	4.86	3.57
		sd	±1.85	±1.30	±2.08	±1.40
FES- natural	4.99	±1.85		$<<10^{-16}$	0.003	$<<10^{-16}$
FES- cryo-damaged	3.30	±1.30	1.46			$<<10^{-16}$
FES+ natural	4.86	±2.08	0.07			$<<10^{-16}$
FES+ cryo-damaged	3.57	±1.40	0.83	0.20	0.74	

p-avlue

Cohen´s D

Supplementary Figure S4: Axon diameter (AD). The AD is given here in µm as mean ± sd. Statistical significance is assumed for p-values of $p < 0.05$. The Cohen's D is interpreted as small for values around $d = 0.2$, as medium for values around $d = 0.5$ and as large for values around 0.8 and larger. The AD values are given for the natural and cryo-damaged RLNs without functional electrical stimulation (FES^{-}), as well as for the natural and cryo-damaged RLNs with functional electrical stimulation (FES^{+}).

S5 – Muscle thickness statistics

MT				FES-		FES+	
				natural	cryo-damaged	natural	cryo-damaged
		mean		2.41	1.96	2.31	1.80
			sd	±0.72	±0.81	±0.80	±0.70
FES-	natural	2.41	±0.72		$<<10^{-16}$	$<<10^{-9}$	$<<10^{-16}$
FES-	cryo-damaged	1.96	±0.81	0.60			$<<10^{-16}$
FES+	natural	2.31	±0.80	0.14			$<<10^{-16}$
FES+	cryo-damaged	1.80	±0.70	0.86	0.21	0.68	

p-avlue (upper right triangle); Cohen´s D (lower left triangle)

Supplementary Figure S5: Myelin thickness (MT). The MT is given here in µm as mean ± sd. Statistical significance is assumed for p-values of $p < 0.05$. The Cohen's D is interpreted as small for values around $d = 0.2$, as medium for values around $d = 0.5$ and as large for values around 0.8 and larger. The MT values are given for the natural and cryo-damaged RLNs without functional electrical stimulation (FES^-), as well as for the natural and cryo-damaged RLNs with functional electrical stimulation (FES^+).

S6 – Overall nerve diameter statistics

OD				FES-		FES+	
				natural	cryo-damaged	natural	cryo-damaged
		mean		9.81	7.21	9.48	7.17
			sd	±2.38	±1.84	±2.77	±1.82
FES-	natural	9.81	±2.38		$\ll 10^{-16}$	$\ll 10^{-8}$	$\ll 10^{-16}$
FES-	cryo-damaged	7.21	±1.84	1.21			0.24
FES+	natural	9.48	±2.77	0.02			$\ll 10^{-16}$
FES+	cryo-damaged	7.17	±1.82	1.20	0.13	1.00	

p-avlue

Cohen´s D

Supplementary Figure S6: Overall diameter (OD). The OD is given here in µm as mean ± sd. Statistical significance is assumed for p-values of $p < 0.05$. The Cohen's D is interpreted as small for values around $d = 0.2$, as medium for values around $d = 0.5$ and as large for values around 0.8 and larger. The OD values are given for the natural and cryo-damaged RLNs without functional electrical stimulation (FES^-), as well as for the natural and cryo-damaged RLNs with functional electrical stimulation (FES^+).

S7 – Fibre type ratio statistics

FTR				FES$^-$						FES$^+$					
				CT		SHAM				DC04				DC07	
				nPCA$^-$	cdPCA$^-$	nPCA$^+$	nPCA^{++}	cdPCA$^+$	cdPCA^{++}	nPCA$^+$	nPCA^{++}	cdPCA$^+$	cdPCA^{++}	nPCA$^+$	nPCA^{++}	cdPCA$^+$	cdPCA^{++}
		mean		1.25	1.50	1.53	1.30	3.21	2.70	13.75	49.00	4.05	3.75	4.43	10.97	17.48	11.07
			sd	±0.21	±0.57	±0.6	±0.57	±2.26	±2.14	±11.75	±32.94	±2.29	±1.46	±2.07	±5.78	±20.79	±12.94
FES$^-$ CT	nPCA$^-$	1.25	±0.21		0.14	0.48	0.93	0.24	0.29	0.14	0.08	0.13	0.08	0.08	0.08	0.24	0.14
FES$^-$ CT	cdPCA$^-$	1.50	±0.57	0.57				0.31	0.36			0.14	0.08			0.24	0.14
FES$^-$ SHAM	nPCA$^+$	1.53	±0.6	0.55			0.36	0.14		0.01				0.05			
FES$^-$ SHAM	nPCA^{++}	1.30	±0.57	0.10		0.39			0.01		0.01				0.01		
FES$^-$ SHAM	cdPCA$^+$	3.21	±2.26	1.03	0.89	0.98			0.75			0.52				0.14	
FES$^-$ SHAM	cdPCA^{++}	2.70	±2.14	0.81	0.66		0.89	0.23					0.44				0.08
FES$^+$ DC04	nPCA$^+$	13.75	±11.75	1.50		1.80					$\ll 10^{-3}$	0.03		0.24			
FES$^+$ DC04	nPCA^{++}	49.00	±32.94	2.29			3.07			1.55			$\ll 10^{-2}$		0.14		
FES$^+$ DC04	cdPCA$^+$	4.05	±2.29	1.72	1.53			0.37		1.15			0.61			0.33	
FES$^+$ DC04	cdPCA^{++}	3.75	±1.46	2.67	2.20				0.52		1.94	0.15					0.34
FES$^+$ DC07	nPCA$^+$	4.43	±2.07	2.16		2.24				1.10					0.08	0.18	
FES$^+$ DC07	nPCA^{++}	10.97	±5.78	2.38			3.02				1.78			1.51			0.90
FES$^+$ DC07	cdPCA$^+$	17.48	±20.79	0.99	0.97			1.05				0.81		0.79			0.78
FES$^+$ DC07	cdPCA^{++}	11.07	±12.94	1.00	0.98				1.05				0.69		0.01	0.36	

q-value

Cohen´s D

Supplementary Figure S7: Fibre Type Ratio (FTR). The FTR is given here in relative units as mean ± sd. Statistical significance is assumed for q-values of $q < 0.1$. The Cohen's D is interpreted as small for values around $d = 0.2$, as medium for values around $d = 0.5$ and as large for values around 0.8 and larger. The HF values are given for the groups without functional electrical stimulation (FES$^-$): control group (CT) and the group with electrode implantation (SHAM), as well as for the groups with functional electrical stimulation (FES$^+$): electrode implantation and DC of 40% (DC04) and the group with electrode implantation and DC of 70% (DC07). All groups consist of a PCA side with a natural RLN (nPCA) and a PCA side with cryo-damaged RLN (cdPCA).

S8 – Slow muscle fiber diameter statistics

Slow MFD					FES$^-$						FES$^+$							
					CT		SHAM				DC04				DC07			
					nPCA$^-$	cdPCA$^-$	nPCA$^+$	nPCA^{++}	cdPCA$^+$	cdPCA^{++}	nPCA$^+$	nPCA^{++}	cdPCA$^+$	cdPCA^{++}	nPCA$^+$	nPCA^{++}	cdPCA$^+$	cdPCA^{++}
			mean		27.2	28.7	25.8	27.8	26.8	28.9	26.6	28.8	26.9	34.0	27.6	30.6	25.2	28.7
				sd	±4.2	±3.5	±3.8	±3.8	±2.8	±4.9	±3.2	±5.0	±3.5	±2.5	±4.0	±4.7	±3.7	±3.2
FES$^-$	CT	nPCA$^-$	27.2	±4.2		0.006	0.84	0.95	0.95	0.84	0.95	0.88	0.95	0.19	0.95	0.72	0.84	0.84
		cdPCA$^-$	28.7	±3.5	0.40				0.72	0.95			0.84	0.24			0.46	0.99
	SHAM	nPCA$^+$	25.8	±3.8	0.35			$<<10^{-13}$	$<<10^{-4}$		0.88				0.84			
		nPCA^{++}	27.8	±3.8	0.15		0.52			$<<10^{-5}$		0.88				0.71		
		cdPCA$^+$	26.8	±2.8	0.10	0.62	0.32			$<<10^{-14}$			0.99				0.77	
		cdPCA^{++}	28.9	±4.9	0.37	0.04		0.27	0.53					0.72				0.84
FES$^+$	DC04	nPCA$^+$	26.6	±3.2	0.15		0.23					$<<10^{-13}$	0.24		0.88			
		nPCA^{++}	28.8	±5.0	0.35			0.24			0.53			0.01		0.88		
		cdPCA$^+$	26.9	±3.5	0.08	0.53			0.01		0.07			$<<10^{-14}$			0.84	
		cdPCA^{++}	34.0	±2.5	1.89	1.69				1.15		1.34	2.27					0.84
	DC07	nPCA$^+$	27.6	±4.0	0.10		0.47				0.27					$<<10^{-4}$	$<<10^{-14}$	
		nPCA^{++}	30.6	±4.7	0.76			0.70				0.38			0.68			$<<10^{-4}$
		cdPCA$^+$	25.2	±3.7	0.51	0.97			0.50				0.38		0.63			$<<10^{-14}$
		cdPCA^{++}	28.7	±3.2	0.43	0.00				0.05				1.76		0.49	1.02	

q-value

Cohen´s D

Supplementary Figure S8: Slow fibre minimal diameter (slow MFD). The slow MFD is given here in µm as mean ± sd. Statistical significance is assumed for q-values of $q < 0.1$. The Cohen's D is interpreted as small for values around $d = 0.2$, as medium for values around $d = 0.5$ and as large for values around 0.8 and larger. The HF values are given for the groups without functional electrical stimulation (FES$^-$): control group (CT) and the group with electrode implantation (SHAM), as well as for the groups with functional electrical stimulation (FES$^+$): electrode implantation and DC of 40% (DC04) and the group with electrode implantation and DC of 70% (DC07). All groups consist of a PCA side with a natural RLN (nPCA) and a PCA side with cryo-damaged RLN (cdPCA).

S9 – Fast muscle fiber diameter statistics

Fast MFD					FES$^-$						FES$^+$							
					CT		SHAM				DC04				DC07			
					nPCA$^-$	cdPCA$^-$	nPCA$^+$	nPCA^{++}	cdPCA$^+$	cdPCA^{++}	nPCA$^+$	nPCA^{++}	cdPCA$^+$	cdPCA^{++}	nPCA$^+$	nPCA^{++}	cdPCA$^+$	cdPCA^{++}
			mean		32.1	33.9	31.7	34.8	31.3	32.4	31.4	31.7	30.7	34.0	31.5	32.5	29.1	30.3
				sd	±2.7	±3.5	±3.4	±3.6	±4.5	±4.1	±2.8	±7.3	±3.8	±3.1	±5.6	±6.3	±5.0	±7.2
FES$^-$	CT	nPCA$^-$	32.1	±2.7		0.005	0.98	0.75	0.98	0.98	0.98	0.98	0.98	0.82	0.98	0.98	0.77	0.98
		cdPCA$^-$	33.9	±3.5	0.57				0.77	0.98			0.77	0.98			0.53	0.82
	SHAM	nPCA$^+$	31.7	±3.4	0.11			$\ll 10^{-14}$	0.002		0.98				0.98			
		nPCA^{++}	34.8	±3.6	0.82		0.88			$\ll 10^{-5}$		0.77				0.82		
		cdPCA$^+$	31.3	±4.5	0.20	0.61	0.12			0.18			0.98				0.90	
		cdPCA^{++}	32.4	±4.1	0.09	0.36		0.62	0.27					0.98				0.77
FES$^+$	DC04	nPCA$^+$	31.4	±2.8	0.26		0.12					0.98	0.54		0.98			
		nPCA^{++}	31.7	±7.3	0.08			0.67			0.06			0.77		0.98		
		cdPCA$^+$	30.7	±3.8	0.42	0.87			0.13		0.20			$\ll 10^{-5}$			0.98	
		cdPCA^{++}	34.0	±3.1	0.65	0.03				0.39		0.41	0.93					0.98
	DC07	nPCA$^+$	31.5	±5.6	0.14		0.06				0.03					0.75	0.01	
		nPCA^{++}	32.5	±6.3	0.09			0.05				0.13			0.18			0.18
		cdPCA$^+$	29.1	±5.0	0.70	1.07			0.46				0.35		0.46			0.98
		cdPCA^{++}	30.3	±7.2	0.30	0.58				0.38				0.58		0.32	0.19	

q-value

Cohen´s D

Supplementary Figure S9: Fast fibre minimal diameter (fast MFD). The fast MFD is given here in µm as mean ± sd. Statistical significance is assumed for q-values of q < 0.1. The Cohen's D is interpreted as small for values around d = 0.2, as medium for values around d = 0.5 and as large for values around 0.8 and larger. The HF values are given for the groups without functional electrical stimulation (FES$^-$): control group (CT) and the group with electrode implantation (SHAM), as well as for the groups with functional electrical stimulation (FES$^+$): electrode implantation and DC of 40% (DC04) and the group with electrode implantation and DC of 70% (DC07). All groups consist of a PCA side with a natural RLN (nPCA) and a PCA side with cryo-damaged RLN (cdPCA).

S10 – Hybrid fibre statistics

HF				CT $nPCA^{-}$	CT $cdPCA^{-}$	SHAM $nPCA^{+}$	SHAM $nPCA^{++}$	SHAM $cdPCA^{+}$	SHAM $cdPCA^{++}$	DC04 $nPCA^{+}$	DC04 $nPCA^{++}$	DC04 $cdPCA^{+}$	DC04 $cdPCA^{++}$	DC07 $nPCA^{+}$	DC07 $nPCA^{++}$	DC07 $cdPCA^{+}$	DC07 $cdPCA^{++}$
				FES^{-}						FES^{+}							
		mean		1.56	5.90	1.37	3.57	3.59	2.50	3.94	1.77	2.89	13.32	3.56	10.16	3.16	4.60
			sd	±0.39	±3.41	±1.06	±2.85	±2.46	±1.81	±4.21	±1.91	±1.25	±14.70	±3.03	±11.77	±2.15	±5.43
FES^{-}	CT	$nPCA^{-}$	1.56 ±0.39		0.002	0.89	0.42	0.34	0.42	0.43	0.60	0.42	0.42	0.42	0.31	0.42	0.42
FES^{-}	CT	$cdPCA^{-}$	5.90 ±3.41	1.79				0.45	0.34			0.42	0.43			0.42	0.72
FES^{-}	SHAM	$nPCA^{+}$	1.37 ±1.06	0.20			0.34	0.17		0.42				0.42			
FES^{-}	SHAM	$nPCA^{++}$	3.57 ±2.85	0.84		0.99			0.42		0.43				0.36		
FES^{-}	SHAM	$cdPCA^{+}$	3.59 ±2.46	0.99	0.83	1.14			0.38			0.31				0.44	
FES^{-}	SHAM	$cdPCA^{++}$	2.50 ±1.81	0.62	1.41		0.45	0.51					0.42				0.43
FES^{+}	DC04	$nPCA^{+}$	3.94 ±4.21	0.80		0.99					0.78	0.34		0.86			
FES^{+}	DC04	$nPCA^{++}$	1.77 ±1.91	0.17			0.67			0.62			0.43		0.43		
FES^{+}	DC04	$cdPCA^{+}$	2.89 ±1.25	1.43	1.17			0.32		0.34			0.34			0.86	
FES^{+}	DC04	$cdPCA^{++}$	13.32 ±14.70	1.26	0.77				1.52		1.10	1.12					0.42
FES^{+}	DC07	$nPCA^{+}$	3.56 ±3.03	0.93		1.12				0.10					0.42	0.66	
FES^{+}	DC07	$nPCA^{++}$	10.16 ±11.77	1.03			0.96				0.91			0.77			0.43
FES^{+}	DC07	$cdPCA^{+}$	3.16 ±2.15	0.93	1.02			0.19				0.14		0.16			0.55
FES^{+}	DC07	$cdPCA^{++}$	4.60 ±5.43	0.71	0.27				0.56				0.96		0.66	0.35	

q-value (upper triangle); Cohen´s D (lower triangle)

Supplementary Figure S10: Hybrid fibres (HF). The HF are given here in % as mean ± sd. Statistical significance is assumed for q-values of $q < 0.1$. The Cohen's D is interpreted as small for values around $d = 0.2$, as medium for values around $d = 0.5$ and as large for values around 0.8 and larger. The HF values are given for the groups without functional electrical stimulation (FES^{-}): control group (CT) and the group with electrode implantation (SHAM), as well as for the groups with functional electrical stimulation (FES^{+}): electrode implantation and DC of 40% (DC04) and the group with electrode implantation and DC of 70% (DC07). All groups consist of a PCA side with a natural RLN (nPCA) and a PCA side with cryo-damaged RLN (cdPCA).

S11 – Collagen amount statistics

Collagen amount				FES^{-}						FES^{+}							
				CT		SHAM				DC04				DC07			
				nPCA^{-}	cdPCA^{-}	nPCA^{+}	nPCA^{++}	cdPCA^{+}	cdPCA^{++}	nPCA^{+}	nPCA^{++}	cdPCA^{+}	cdPCA^{++}	nPCA^{+}	nPCA^{++}	cdPCA^{+}	cdPCA^{++}
		mean		34.0	31.2	33.7	30.4	35.8	30.6	32.1	31.5	29.1	28.8	31.6	29.5	36.3	33.2
			sd	±5.7	±3.3	±3.8	±8.0	±2.9	±5.2	±7.2	±14.3	±5.2	±6.9	±0.7	±13.4	±4.8	±10.7
FES^{-} CT	nPCA^{-}	34.0	±5.7		0.384	0.94	0.94	0.94	0.77	0.94	0.94	0.769	0.778	0.805	0.943	0.38	0.94
FES^{-} CT	cdPCA^{-}	31.2	±3.3	0.60				0.38	0.94			0.94	0.94			0.46	0.94
FES^{-} SHAM	nPCA^{+}	33.7	±3.8	0.07			0.94	0.77		0.94				0.77			
FES^{-} SHAM	nPCA^{++}	30.4	±8.0	0.49		0.51			0.94		0.94				0.94		
FES^{-} SHAM	cdPCA^{+}	35.8	±2.9	0.46	1.53	0.63			0.29			0.292				0.94	
FES^{-} SHAM	cdPCA^{++}	30.6	±5.2	0.63	0.12		0.03	1.23					0.94				0.84
FES^{+} DC04	nPCA^{+}	32.1	±7.2	0.29		0.31					0.77	0.29		0.94			
FES^{+} DC04	nPCA^{++}	31.5	±14.3	0.25			0.11			0.06			0.77		0.94		
FES^{+} DC04	cdPCA^{+}	29.1	±5.2	0.91	0.49			1.81		0.48			0.94			0.38	
FES^{+} DC04	cdPCA^{++}	28.8	±6.9	0.84	0.47				0.32		0.24	0.04					0.94
FES^{+} DC07	nPCA^{+}	31.6	±0.7	0.60		0.68				0.11					0.95	0.46	
FES^{+} DC07	nPCA^{++}	29.5	±13.4	0.44			0.09				0.14			0.22			0.95
FES^{+} DC07	cdPCA^{+}	36.3	±4.8	0.45	1.18			0.14				1.46		1.23			0.77
FES^{+} DC07	cdPCA^{++}	33.2	±10.7	0.09	0.22				0.11				0.44		0.31	0.38	

q-value

Cohen´s D

Supplementary Figure S11: Collagen amount. The Collagen amount is given here in % as mean ± sd. Statistical significance is assumed for q-values of $q < 0.1$. The Cohen’s D is interpreted as small for values around $d = 0.2$, as medium for values around $d = 0.5$ and as large for values around 0.8 and larger. The collagen amount is given for the groups without functional electrical stimulation (FES^{-}): control group (CT) and the group with electrode implantation (SHAM), as well as for the groups with functional electrical stimulation (FES^{+}): electrode implantation and DC of 40% (DC04) and the group with electrode implantation and DC of 70% (DC07). All groups consist of a PCA side with a natural RLN (nPCA) and a PCA side with cryo-damaged RLN (cdPCA).
